# The Extent of the Use of Multivitamins and Multimineral Supplements Without Clinically Measurable Benefits Among Adults in Ha'il, Saudi Arabia: A Cross-Sectional Study

**DOI:** 10.7759/cureus.38750

**Published:** 2023-05-09

**Authors:** Amany M Khalifa, Zahwah D Alshammari, Afnan A Altamimi, Areeb Alshammari

**Affiliations:** 1 Parasitology, Alexandria University, Alex, EGY; 2 Pathology-Parasitology, University of Ha'il, Ha'il, SAU; 3 Medicine and Surgery, University of Ha'il College of Medicine, Ha'il, SAU

**Keywords:** ha’il, saudi arabia, nutritional supplements, multivitamins, multimineral

## Abstract

Introduction

Multivitamins and multiminerals (MVMM) are nutritional supplements that contain a wide range of important nutrients. The use of vitamins and minerals has been showing a tremendous increase over the past few years due to the high demand for supplements to replenish nutritional deficiencies.

Purpose

This study aimed to assess MVMM usage, the reasons why people chose to use MVMM, and the factors related to this usage.

Methodology

A cross-sectional study was conducted on adults living in Ha'il, Saudi Arabia. Data were collected between October 31 and December 14, 2022, a self-administered online questionnaire was used, and data were analyzed using SPSS version 25.0 (IBM Corp, Armonk, NY).

Results

A total of 310 participants enrolled in the study, of which 240 (77.42%) were females and 70 (22.58%) were males. The extent of the use of MVMM supplements without clinically measurable benefits was more than half of the present study participants (58.71%). There was a significant difference between MVMM use and gender or employment status. MVMM usage on a regular basis was found to be associated with satisfaction with the outcomes. The majority of participants used MVMM to promote health. Calcium and vitamin D were found to be the most common types of dietary supplements used.

Conclusion

The use of MVMM supplements without clinically measurable benefits was more prevalent among females. It is important to promote public health awareness programs about the benefits and risks of overdose.

## Introduction

Multivitamins and multiminerals (MVMM) are described as dietary supplements, which are different from ordinary food and are meant to enhance or complement the diet. Even if a product is labeled as a dietary supplement, it is still regarded as a drug, to the extent that it is meant to treat, diagnose, cure, or prevent diseases [1]. Capsules, tablets, soft gels, gummies, and liquid supplements are just some of the many various forms that supplements can be ingested. Such examples of these supplements are vitamins that can be multiple or single such as vitamin D and biotin. Also, minerals are supplements such as calcium and iron. Herbs also can be considered supplements such as ginger and echinacea [1,2]. An overwhelming amount of physiological data demonstrates the essential role of vitamins and minerals in energy metabolism, primarily, the B complex vitamins, which are required for mitochondrial function, thus, a deficiency of any one of these vitamins can jeopardize an entire chain of biochemical events required for converting food into physiological energy [3].

Despite a well-balanced and generally healthy diet, vitamin deficiencies can occur occasionally, which can have an impact on the individual’s health [4]. Specific individual requirements vary depending on health, lifestyle, genetics, and other variables, hindering these needs challenging to quantify [3]. Nonetheless, using MVMM supplements has been found to minimize food intake gaps and enhance nutritional status without exceeding the dietary reference intake (DRI) [5]. 

Recently, with the COVID-19 pandemic, the use of MVMM such as vitamin D and vitamin C has been prevalent with evidence of improving the outcome of various respiratory infections [6]; regardless, a huge gap is present in the literature regarding the benefits of MVMM in COVID-19 patients. However, it is important to note that MVMM do not have a positive impact on all diseases, many randomized clinical studies have failed to support the idea that multivitamins can prevent chronic diseases specifically cardiovascular diseases such as stroke or myocardial infarction [7-9]. Even so, several studies show that using MVMM in levels more than the recommended daily intake (RDA), for example, large dosages of folic acid, beta carotene, vitamin E, and selenium may be detrimental, increasing mortality and leading to cancer in some patients [4,8].

The use of MVMM is often associated with adopting many healthy lifestyle habits [2,10,11]; however, understanding the health influences of consuming such preparations is crucial, especially in the absence of clinical research carried out by dietary supplement manufacturers prior to their products being introduced to the market.

Dietary supplements are not indicated for general health support and illness prevention, but rather for people who have a chronic nutritional shortage in their diet or a previously confirmed deficiency in their body [4]. As a result, greater focus should be placed on dietary adjustments, such as the advantages of eating more fruits and vegetables, where vitamins and minerals occur naturally in conjunction with other elements that cannot be mimicked by food supplements [12]. Yet, people continue to use the dietary supplements on their own without consultation which might increase the risk of toxicities, drug overdose, and consequent health problems [5,10]. Our study aimed to estimate the extent of MVMM users in Ha'il City, Saudi Arabia. And assess the pattern of the usage, its association with their sociodemographic data, and their general health status. Moreover, to investigate the factors, which justify using MVMM without actual clinical significance.

## Materials and methods

This cross-sectional study was carried out in Ha'il City, Saudi Arabia. For our inclusion criteria, participants were required to be 18 years or older, living in Ha'il City, and willing to participate in the study. We excluded incomplete submissions. Participants were reached through multiple social media platforms, such as Twitter, WhatsApp, etc., and were asked to fill out an electronic Google Form questionnaire (Appendices). Participation was voluntary and withdrawal was available at any time. A pilot questionnaire was conducted previously to measure comprehension and clarity. We received 310 responses, which demonstrated a complete understanding of the questions.

Data were collected between October 31 to December 14, 2022. The first section of the questionnaire covered the sociodemographics of the participants, including age, gender, nationality, residence, educational level, physical activity, family income, and relationship status. The second section included items addressing the use, frequency, and supply of MVMM. As well as checking participants' current use of MVMM, what specific types of MVMM are used, and participants' satisfaction after using these MVMM. The third section covered information regarding why participants used MVMM, who prescribed these MVMM, and participants spending budget on MVMM. Lastly, participants were asked to rate their overall health using a four-point Likert scale (excellent/very good, good, fair, poor), state if they have any chronic conditions - and how many if answered yes, and whether they are smoking or not.

Sample size

The sample size ideal for conducting this cross-sectional study was 273, which was calculated using the following formula: SS = [Z2p (1 − p)]/ C2. Where Z is 1.96, C is 5%, SS is the population of the Ha'il region, and p is 77% according to a recent study regarding prevalence of MVMM use in Saudi Arabia [13]. In total, 310 participants were included in the study.

## Results

Data in this study were analyzed using SPSS version 25.0 for Windows (IBM Corp., Armonk, NY). Internal consistency was used to test the validation of the scale. The frequencies were conducted to describe the items. The chi-square test was used to test the cross relationships between the variables. A p-value of more than 0.05 was considered statistically significant.

As shown in Table 1, 310 people participated in the study, including 77.42% females and 22.58% males, most of them were in the young age group (18-29). The majority were Saudis, lived in Hail City, were university educated, 42.9% had a family monthly income of more than 10.000 SAR, and 57.42% were single. Only 20% were health professionals and 58.71% of the study participants used a MVMM.

**Table 1:** Sociodemographic Information (N=310)

Factor		N	%
Gender	Male	70	22.58
	Female	240	77.42
Age	18-29	181	58.39
	30-39	58	18.71
	40-49	44	14.19
	>=50	27	8.71
Nationality	Saudi	306	98.71
	Non-Saudi	4	1.29
Residency	Hail City	250	80.65
	Outside Hail City	60	19.35
Family income relative	<5000 SAR	59	19.03
	5000-10000 SAR	118	38.06
	>10000 SAR	133	42.90
Education status	Primary school	2	0.65
	Middle school	7	2.26
	Secondary school	56	18.06
	University	245	79.03
Relationship status	Single	178	57.42
	Married	124	40.00
	Divorced, or widowed	8	2.58
Employment status	Health professional	62	20.00
	Other	171	55.16
	Unemployed	77	24.84
Do you use a multivitamin and/or multimineral currently?	Yes	182	58.71
	No	128	41.29

**Figure 1:**
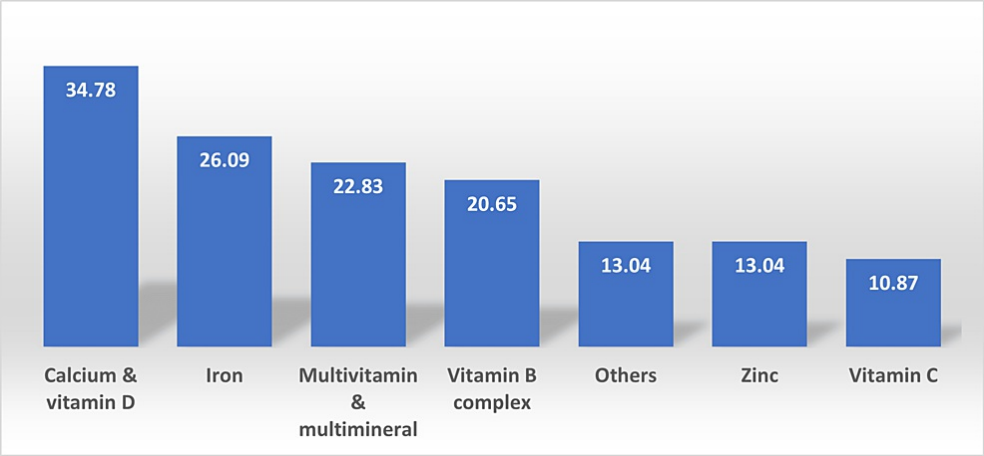
Frequency of Vitamins and Minerals Taken

Table 2 showed that 46.7% of the study participants reported good health, followed by excellent/very good health, and 23.08% had fair health. Also, 76.92% had no chronic conditions while 65.93% did moderate physical activity. Forty-one point seventy-six percent (41.76%) of the study participants used MVMM usage on a regular basis. Forty-eight point nine percent (48.9%) took these products via hospitals as a prescription. Prompting health was the most reason for using these products with 38.71%, followed by a supplement diet. Forty-one point two one (41.21%) used to take MVMM daily, followed by weekly usage. Physicians were the source of information with 50.55%, followed by media and the Internet. Also, most of the participants reported that the usage of MVMM should be used with a medical prescription while 14.84% argued that they should be used on believing that diet covers all the nutrients needed. Sixty-nine point two three percent (69.23%) spent less than 200 Saudi Arabian Riyals (SAR) on supplements. Seventy-six point nine two percent (76.92%) addressed their satisfaction with the outcomes of using supplements. Thirty-four point seven eight percent (34.78%) of the present study participants preferred to take calcium and vitamin D, followed by iron then MVMM. Vitamin B complex was the most used vitamin with 20.65%, followed by others (Figure 1).

**Table 2:** Frequency, Supply, and Usage Reasons for MVMM (N=182) MVMM: multivitamins and multiminerals

Factor	N	%	
Self-rated overall health	Poor	3	1.65
	Fair	42	23.08
	Good	85	46.70
	Excellent/very good	52	28.57
History of chronic conditions	No chronic conditions	140	76.92
	One chronic condition	31	17.03
	Multiples chronic conditions	11	6.04
Physical activity	None	55	30.22
	Moderate	120	65.93
	Vigorous	7	3.85
Cigarette smoking	Yes	11	6.04
	No	171	93.96
Multivitamin and Multimineral Supplement (MVMM) Usage on regular basis:	Yes	76	41.76
	No	106	58.24
Sources of these products	Hospitals as prescription	89	48.90
	Pharmacy as over the counter	57	31.32
	Health/supplements stores	9	4.95
	Websites/social media	16	8.79
	Friends and family	11	6.04
Why are you using these products?	Supplement diet	110	35.48
	To promote health	120	38.71
	To treat a disease	46	14.84
	Enhance Physical appearance	50	16.13
	To prevent a disease	44	14.19
Frequency of using multivitamins and multimineral (MVMM)	Daily	75	41.21
	Weekly	65	35.71
	Monthly	42	23.08
Sources of information regarding multivitamins	Physician	92	50.55
	Family and friends	30	16.48
	Media & internet	60	32.97
Advise the usage of dietary supplements and multivitamins	Yes, with a medical prescription	149	81.87
	Believe that diet covers all the nutrients needed	27	14.84
	Warn against the usage of supplements	6	3.30
Money spends on supplement	< 200 SAR	126	69.23
	200 - 500 SAR	49	26.92
	> 500 SAR	7	3.85
Are you satisfied with the outcomes of using supplements?	Yes	140	76.92
	No	42	23.08

As shown in Table 3, there were no significant relationships between MVMM usage on a regular basis and sociodemographic factors (p>0.05).

**Table 3:** The Relationship Between MVMM Usage on a Regular Basis and Sociodemographic Information (N=182) MVMM: multivitamins and multiminerals

Factor		Multivitamin and Multimineral Supplement (MVMM) Usage on a Regular Basis				X^2^	p
		Yes		No			
		N	%	N	%		
Gender	Male	10	31.25	22	68.75	1.76(ns)	0.18
	Female	66	44.00	84	56.00		
Age	18-29	41	40.20	61	59.80	4.99(ns)	0.17
	30-39	11	30.56	25	69.44		
	40-49	13	56.52	10	43.48		
	>=50	11	52.38	10	47.62		
Nationality	Saudi	76	42.46	103	57.54	2.18(ns)	0.13
	Non-Saudi	0	0.00	3	100.00		
Residency	Hail City	64	42.95	85	57.05	0.48(ns)	0.48
	Outside Hail City	12	36.36	21	63.64		
Family income relative	<5000 SAR	13	39.39	20	60.61	4.45(ns)	0.10
	5000-10000 SAR	25	33.78	49	66.22		
	>10000 SAR	38	50.67	37	49.33		
Education status	Primary school	2	100.00	0	0.00	3.79(ns)	0.28
	Middle school	3	60.00	2	40.00		
	Secondary school	15	44.12	19	55.88		
	University	56	39.72	85	60.28		
Relationship status	Single	42	41.18	60	58.82	0.25(ns)	0.88
	Married	32	43.24	42	56.76		
	Divorced, or widowed	2	33.33	4	66.67		
Employment status	Health professional	16	44.44	20	55.56	1.88(ns)	0.39
	Other	34	36.96	58	63.04		
	Unemployed	26	48.15	28	51.85		
*≤0.05; **≤0.01; ** ≤0.001; ns=Not significant							

Two interesting relationships were found across the results as shown in Table 4. There was a significant relationship between MVMM usage on a regular basis and satisfaction with the outcomes of using supplements (p>0.05; =0.02). Eighty-six point six seven (86.67%) of participants using MVMM on a regular basis were satisfied with the outcomes of using supplements compared to those who did not use it regularly, at 70.75%. Also, 32.86% who had excellent/very good health were satisfied as compared to 14.29% who were not satisfied. In addition, 50% who had good health were satisfied as compared to 35.71% who were not satisfied (p>001).

**Table 4:** The Relationship Between MVMM Usage, Satisfaction, and Overall Health (N=182) MVMM: multivitamins and multiminerals

Multivitamin and Multimineral Supplement (MVMM) Usage on a Regular Basis		Yes		No		X^2^	p
		N	%	N	%		
Satisfaction with the outcomes of using supplements	Yes	65	86.67	75	70.75	5.44*	0.020
	No	11	14.67	31	29.25		
Frequency of using multivitamins and multimineral (MVMM)	Daily	48	64.00	27	25.47	27.39***	<0.001
	Weekly	20	26.67	45	42.45		
	Monthly	8	10.67	34	32.08		
Satisfaction with the outcomes of using supplements		Yes		No		X^2^	p
		N	%	N	%		
Self-rated overall health	Poor	1	0.71	2	4.76	20.14***	<0.001
	Fair	23	16.43	19	45.24		
	Good	70	50.00	15	35.71		
	Excellent/very good	46	32.86	6	14.29		
*≤0.05; **≤0.01; ** ≤0.001; ns=Not significant							

As shown in Table 5, two relationships between MVMM usage and sociodemographic factors were found (p>0.05). Sixty-two point five percent (62.5%) of females used MVMM as compared to males (p<0.05), and 70.13% of unemployed used MVMM as compared to health professionals (58.06%) and others (53.80%) (p<0.05).

**Table 5:** The Relationship Between MVMM Usage and Sociodemographic Information (N=310) MVMM: multivitamins and multiminerals

Factor		Do you use a multivitamin and/or multimineral currently?				X^2^	p
		Yes		No			
		N	%	N	%		
Gender	Male	32	45.71	38	54.29	6.30*	0.011
	Female	150	62.50	90	37.50		
Age	18-29	102	56.35	79	43.65	5.49	0.14
	30-39	36	62.07	22	37.93		
	40-49	23	52.27	21	47.73		
	>=50	21	77.78	6	22.22		
Nationality	Saudi	179	58.50	127	41.50	0.44	0.51
	Non-Saudi	3	75.00	1	25.00		
Residency	Hail City	149	59.60	101	40.40	0.42	0.52
	Outside Hail City	33	55.00	27	45.00		
Family income relative	<5000 SAR	33	55.93	26	44.07	1.26	0.53
	5000-10000 SAR	74	62.71	44	37.29		
	>10000 SAR	75	56.39	58	43.61		
Education status	Primary school	2	100.00	0	0.00	2.10	0.55
	Middle school	5	71.43	2	28.57		
	Secondary school	34	60.71	22	39.29		
	University	141	57.55	104	42.45		
Relationship status	Single	102	57.30	76	42.70	1.07	0.59
	Married	74	59.68	50	40.32		
	Divorced, or widowed	6	75.00	2	25.00		
Employment status	Health professional	36	58.06	26	41.94	6.00*	0.05
	Other	92	53.80	79	46.20		
	Unemployed	54	70.13	23	29.87		
*≤0.05; **≤0.01; ** ≤0.001; ns=Not significant							

## Discussion

In Saudi Arabia, vitamins and mineral supplements are used extensively. However, there’s a lack of studies investigating MVMM usage among Saudi Arabia’s population and the factors related to it. Furthermore, most studies focused only on one or two dietary supplements [14-16], disregarding MVMM usage, which is what this study is targeting. Our study of 310 participants showed a significant positive relationship between MVMM usage with gender and employment status. These results are constant with the findings of other studies [17,18]. One cross-sectional study conducted in the United States of America (USA) demonstrated a higher MVMM prevalence in females as well [19]. In another study in Japan with 1,776 participants, females were far more likely to report supplement use than males [20]. Again, our current study agrees that MVMM use is higher among females than males (82.4% vs 17.6%). Additionally, MVMM use had been reported with higher use among females than males in several local studies [21-23].

Our study also showed a positive association between MVMM use with unemployed. However, a study performed by Alfawaz et al. (2017) reported that occupation was not associated with the frequency of using dietary supplements during pregnancy in Saudi women [24]. The relationship between MVMM use and employment status needs further focused investigation on its related factors to better understand it. Contrary to our expectations, our study did not suggest a significant difference between MVMM use with age, nationality, residency, family income relative, education, and relationship status.

A similar previous study showed the differences in health status and health outcomes between MVMM users and non-users; despite no clinically measured changes in health, MVMM users self-report 30% greater overall health than non-users [19]. A significant association between participants who self-reported excellent or very good health and satisfaction with outcomes as opposed to those who did not was also noted in our study. Furthermore, most of the participants who use MVMM regularly were satisfied with the outcomes of using it, unlike others who did not use it regularly and were not as satisfied. One study explored reasons behind the belief of perceived better health after using MVMM, results pointed to the concept of not getting ill and adopting a healthier lifestyle [25], which can explain why participants might feel that MVMM is the main reason for improved health outcomes.

Many studies reported that the most common reason for using MVMM was to promote health, the majority of individuals utilized MVMM as diet supplements, followed by health promotion [26]. Vitamin D insufficiency is extremely common in Saudi Arabia among all demographic groups and all different regions; it is linked to a number of extra-skeletal, and chronic diseases such as insulin resistance and its associated comorbidities [14]. That explains why most of our study findings indicate that participants preferred to take calcium and vitamin D rather than any other specific supplement.

Finally, several important limitations need to be considered. First, this was a single-center study, which might have led to bias. Second, some participants might have incorrectly reported their information. Third, the study did not evaluate the reasons why some of the participants did not take the MVMM currently. In addition, a larger and more widespread study is required to be conducted on dietary supplement usage to determine the specific factors related to MVMM usage.

Our study has raised multiple issues that need to be investigated further. An area of future research is to look into the widespread use of MVMM supplements for the purpose of COVID-19 prevention, evaluate the long-term effects of MVMM use in overdosing and toxicity, and identify certain associations between vitamin deficiencies and certain diseases.

## Conclusions

Our study found a significant association between MVMM usage and sociodemographic factors - gender and employment status. Furthermore, the use of MVMM on a regular basis was found to be associated with satisfaction-of-MVMM-use outcomes, as well as high self-reported health outcomes. These findings spotlight the extent of the use of MVMM supplements without clinically measurable benefits. As a result, it was vital to throw light on the importance of public health awareness regarding the advantages of MVMM as well as warnings about overdoses and side effects. Hopefully, this will lead to improvements in the population’s lifestyle.

## Appendices

**Table 6:** Study Questionnaire

Informed consent	
A research team from the Faculty of Medicine - University of Hail aims to know the extent of the use of multivitamins and multimineral supplements without clinically measurable benefits among adults in Hail, Saudi Arabia: Cross sectional study. Please read the following information carefully, all information you provide will be treated confidentially and the identity of any participant will not be disclosed. Your participation is completely voluntary and we greatly appreciate it. Main investigator: Prof. Amany Khalifa .sa	Agree
	Disagree
Socio-demographic characteristics of MVMM users	
Gender	Female
	Male
Age	18-29
	30-39
	40-49
	50 & above
Nationality	Saudi
	Non-Saudi
Hail Residence	Hail City
	Outside Hail City
Family income relative	Less than 5000
	5000-10,000
	More than 10,000
Education status	University
	Secondary
	Middle
	Primary
Relationship status	Single
	Married
	Divorced, or widowed
Employment status	Health professional
	Others
	Unemployed
Do you use a multivitamin and/or multimineral currently?	Yes
	No
Frequency, supply, and usage cause of multivitamins and multiminerals (MVMM).	
Self-rated overall health as:	Excellent/very good
	Good
	Fair
	poor
History of chronic conditions	No chronic conditions.
	One chronic condition.
	Multiple chronic conditions
Physical activity	None
	Moderate
	Vigorous
Cigarette smoking	Yes
	No
Multivitamin and Multimieral suppement (MVMM) Usage on regular basis:	Yes
	No
Frequency of using multivitamins and multimineral (MVMM)	Daily
	Weekly
	Monthly
Sources of these products	Hospitals as prescription
	Pharmacy as over the counter
	Health/supplements stores
	Websites/social media
	Friends and family
Why are you using these products? (You can select more than one item)	Supplement diet
	To promote health
	To treat a disease
	Enhance Physical appearance
	To prevent a disease
Sources of information regarding multivitamins:	Physician
	Family and friends
	Media & internet
Advise the usage of dietary supplements and multivitamins:	Yes, with a medical prescription
	Believe that diet covers all the nutrients needed
	Warn against the usage of supplements
Money spend on supplements:	Less than 200 riyals
	Between 200 and 500 riyals
	More than 500 riyals
Are you satisfied with the outcomes of using supplements?	Yes
	No
What are the vitamins and minerals taken	Multivitamin & multimineral
	Calcium & vitamin D
	Vitamin C
	Vitamin B complex
	Iron
	Zinc
	Others

## References

[1] (2023). U.S. Food and Drug Administration. FDA 101: dietary supplements. https://www.fda.gov/consumers/consumer-updates/fda-101-dietary-supplements.

[2] Dickinson A, MacKay D (2014). Health habits and other characteristics of dietary supplement users: a review. Nutr J.

[3] Huskisson E, Maggini S, Ruf M (2007). The role of vitamins and minerals in energy metabolism and well-being. J Int Med Res.

[4] Rautiainen S, Manson JE, Lichtenstein AH, Sesso HD (2016). Dietary supplements and disease prevention - a global overview. Nat Rev Endocrinol.

[5] Biesalski HK, Tinz J (2017). Multivitamin/mineral supplements: rationale and safety - a systematic review. Nutrition.

[6] El Sabbagh E, El-Sayed M, Elbaz T (2022). Vitamins and minerals: a means for surviving the COVID-19 pandemic or just a myth?. J Infect Dev Ctries.

[7] Sesso HD, Christen WG, Bubes V (2012). Multivitamins in the prevention of cardiovascular disease in men. The Physicians' Health Study II randomized controlled trial. JAMA.

[8] Rautiainen S, Gaziano JM, Christen WG (2017). Effect of baseline nutritional status on long-term multivitamin use and cardiovascular disease risk. A secondary analysis of the Physicians’ Health Study II randomized clinical trial. JAMA Cardiol.

[9] Rautiainen S, Lee IM, Rist PM, Gaziano JM, Manson JE, Buring JE, Sesso HD (2015). Multivitamin use and cardiovascular disease in a prospective study of women. Am J Clin Nutr.

[10] Alowais MA, Selim MA (2019). Knowledge, attitude, and practices regarding dietary supplements in Saudi Arabia. J Family Med Prim Care.

[11] Foote JA, Murphy SP, Wilkens LR, Hankin JH, Henderson BE, Kolonel LN (2003). Factors associated with dietary supplement use among healthy adults of five ethnicities: the Multiethnic Cohort Study. Am J Epidemiol.

[12] Wierzejska RE (2021). Dietary supplements-for whom? The current state of knowledge about the health effects of selected supplement use. Int J Environ Res Public Health.

[13] Alsofyani MAA, Al-Essa MHA, Assiri MA (2018). Prevalence of people that using multivitamins supplementation and experiencing a side effect in Saudi Arabia. Egypt J Hosp Med.

[14] Al-Daghri NM (2018). Vitamin D in Saudi Arabia: prevalence, distribution and disease associations. J Steroid Biochem Mol Biol.

[15] Alokail M, Al-Daghri N, Al-Attas O, Yakout S, Aljohani N, Alfawaz H (2015). Dietary products consumption in relation to serum 25-hydroxyvitamin D and selenium level in Saudi children and adults. Int J Clin Exp Med.

[16] Al-Mohaithef M, Alaslani H, Javed NB, Chandramohan S (2021). Folic acid awareness and usage among females at Saudi Electronic University in Jeddah, Saudi Arabia. SAGE Open Med.

[17] Timbo BB, Ross MP, McCarthy PV, Lin CT (2006). Dietary supplements in a national survey: prevalence of use and reports of adverse events. J Am Diet Assoc.

[18] Ervin RB, Wright JD, Kennedy-Stephenson J (1999). Use of dietary supplements in the United States, 1988-94. Vital Health Stat 11.

[19] Paranjpe MD, Chin AC, Paranjpe I (2020). Self-reported health without clinically measurable benefits among adult users of multivitamin and multimineral supplements: a cross-sectional study. BMJ Open.

[20] McNaughton SA, Mishra GD, Paul AA, Prynne CJ, Wadsworth ME (2005). Supplement use is associated with health status and health-related behaviors in the 1946 British birth cohort. J Nutr.

[21] Alfawaz H, Khan N, Alfaifi A (2017). Prevalence of dietary supplement use and associated factors among female college students in Saudi Arabia. BMC Womens Health.

[22] Algaeed HA, AlJaber MI, Alwehaibi AI (2019). General public knowledge and use of dietary supplements in Riyadh, Saudi Arabia. J Family Med Prim Care.

[23] Alqrache A, Mostafa M, Ghabrah O, Ghabrah Z, Kamal N, Ghabrah T, Atta H (2021). Knowledge and patterns of dietary supplement use among students attending King Abdulaziz University in Saudi Arabia: a cross-sectional study. Inquiry.

[24] Alfawaz HA, Khan N, AlOteabi N, Hussain SD, Al-Daghri NM (2017). Factors associated with dietary supplement use in Saudi pregnant women. Reprod Health.

[25] Conner M, Kirk SFL, Cade JE, Barrett JH (2001). Why do women use dietary supplements? The use of the theory of planned behaviour to explore beliefs about their use. Soc Sci Med.

[26] Alwalan SI, Alrasheed AA, Aldossari KK, Al-Zahrani JM, Alshahrani AM, Batais MA, Almigbal TH (2022). Prevalence and characteristics of multivitamin-multimineral (MVMM) use among Saudi populations in Riyadh, Saudi Arabia: a cross-sectional study. Medicine (Baltimore).

